# Trends in Hypertension and Hypertensive Disease Among Active Component U.S. Service Members, 2018–2023

**Published:** 2025-08-20

**Authors:** Shauna L. Stahlman, Lori M. Tantlinger

**Affiliations:** Epidemiology and Analysis Branch, Armed Forces Health Surveillance Division, Public Health Directorate, Defense Health Agency, Silver Spring, MD: Dr. Stahlman; Navy and Environmental Preventive Medicine Unit TWO, Navy and Marine Corps Force Health Protection Command, Medical Corps, U.S. Navy, Norfolk, VA: LCDR Tantlinger

Hypertension, defined as persistent abnormal elevation of blood pressure above 130 / 80 mmHg, is estimated to have affected more than 47% of U.S. adults between 2021 and 2023. ^1 , 2^ Essential hypertension comprises the majority (95%) of hypertension cases and has no identifiable cause, while secondary hypertension stems from underlying medical conditions such as renal or endocrine disorders. ^3 , 4^ As a major risk factor for cardiovascular disease, hypertension can lead to heart and kidney damage if uncontrolled, which highlights the importance of early intervention on modifiable risk factors such as diet and exercise. This study aimed to examine the trend in annual incidence of hypertension and hypertensive disease, as well as the annual percentage of high blood pressure measurements, among active component service members between 2018 and 2023, using data from the Defense Medical Surveillance System (DMSS).

Incident cases of essential hypertension (International Classification of Diseases, 9th Revision [ICD-9] codes 401*; International Classification of Diseases, 10th Revision [ICD-10] codes I10*), secondary hypertension (ICD-9: 405*; ICD-10: I15*), and hypertensive crisis (ICD-10: I16*; no equivalent ICD-9 code) were identified by the presence of a single inpatient or outpatient encounter with a diagnosis listed in any diagnostic position. Hypertensive heart or kidney disease (ICD-9: 402*–404*; ICD-10: I11*–I13*) cases required documentation of an inpatient encounter or at least 2 outpatient encounters within 60 days of each other with the diagnosis listed in the first or second diagnostic position. Periodic Health Assessment (PHA) data were utilized to describe the annual percentages of service members who had 1 or more high blood pressure measurements, among those who had at least 1 recorded blood pressure measurement available. A high blood pressure measurement was defined by systolic blood pressure greater than or equal to (>=) 130 mmHg or diastolic blood pressure greater than or equal to (>=) 80 mmHg.

Incidence of diagnosed essential hypertension increased from 128.2 to 189.1 per 10,000 person-years (2018–2023), with a temporary decrease in 2020 likely related to reduced health care access during the COVID-19 pandemic **(Figure)** . The percentage of service members who had at least 1 recorded high blood pressure measurement increased from 41.5% to 47.4% during the same period, with the largest annual increase occurring between 2019 and 2020. Secondary hypertension decreased from 4.0 per 10,000 person-years (p-yrs) in 2018 to 2.3 per 10,000 p-yrs in 2023 **(Table)** . Hypertensive heart or kidney disease and hypertensive crisis remained stable (averaging 1.5 and 2.8 per 10,000 p-yrs, respectively).

**FIGURE:**
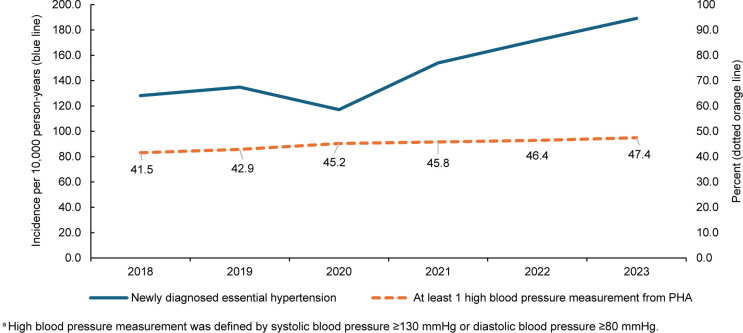
Incidence of Essential Hypertension and Prevalence of High Blood Pressure Measurements ^a^ , Active Component U.S. Service Members, 2018–2023

**TABLE.** Incidence of Hypertension and Other Hypertensive Disease (per 10,000 person-years), Active Component U.S. Service Members, 2018–2023

	2018		2019		2020		2021		2022		2023	
	No.	Rate	No.	Rate	No.	Rate	No.	Rate	No.	Rate	No.	Rate
Essential hypertension	15,567	128.2	16,622	134.8	14,573	117.1	19,255	154.0	20,835	171.8	22,364	189.1
Secondary hypertension	517	4.0	504	3.8	441	3.3	480	3.6	348	2.7	295	2.3
Hypertensive crisis	306	2.4	353	2.7	329	2.5	400	3.0	374	2.9	402	3.2
Hypertensive heart or chronic kidney disease	175	1.4	202	1.5	185	1.4	211	1.6	194	1.5	223	1.8

Abbreviation: No., number.

The increase in essential hypertension among U.S. military personnel is consistent with recent increasing trends of risk factors including obesity and type 2 diabetes, ^5^ and suggests that military fitness requirements alone are insufficient to prevent the development of hypertension. Military members did not, however, show increased rates of more severe hypertensive conditions, possibly indicating protective factors within military health care or lifestyle. In 2017, the definition for high blood pressure was lowered from 140 / 90 mmHg to 130 / 80 mmHg, which raised concerns that increased diagnoses of essential hypertension could be attributed to previously undiagnosed individuals. ^6^ The consistent increase in elevated blood pressure measurements on PHAs suggests a real increase, however, not just more diagnoses occurring under the new guidelines.
